# A Study of the Effect of Cardiac Rehabilitation on Heart Failure Patients’Life Quality

**Published:** 2014-03-25

**Authors:** M Ghanbari-Firoozabadi, AA Rahimianfar, M Reza Vafaii Nasab, SM Namayandeh, M Emami, F Boostani, A Sherafat, K Barzegar

**Affiliations:** * Yazd Cardiovascular Research Center, Shahid Sadoughi University of Medical Sciences, Yazd, Iran; ** Faculty member at the English Language Department, School of Medicine, Shahid Sadoughi University of Medical Sciences, Yazd, Iran

**Keywords:** Cardiac failure, physical activity, rehabilitation, life quality

## Abstract

Abstract

Introduction: Cardiac rehabilitation is a way of improving the quality life of heart failure patients.

Aim: Determining the effect of physical activity on the quality of life of patients with heart failure.

Methods: This study was conducted on 34 patients affected by heart failure with an ejection fraction of less than 40%. The patients followed both psychological and nutritional consultations in the beginning of the program. The patients participated in 24-32 sessions of physical activity three times a week, each time lasting 1-1.5 h. Each session consisted of 20 min of warming up, 20-40 min of aerobic exercises, 5 min of cooling down, and 20 min of relaxation. Physician’s visit, exercise test, echocardiography, fat and blood sugar profile tests were conducted for all patients before and after the rehabilitation program. The Life Quality Form SF-36 was filled out for them and the data were analyzed using the SPSS.

Results: Our findings showed that there was a statistically significant increase in the mean score of the patients’ life quality after rehabilitation. Also, the comparison of the scores of the eight aspects of patients’ life quality before and after rehabilitation revealed that life quality improved in the following directions: physical functioning, limitation of physical health, limitation of psychological health, energy, social functioning, and physical pain. All differences were statistically significant yet, the difference regarding the aspects of psychological health and general well-being were not significant.

Conclusion: Cardiac rehabilitation is effective on improving the life quality of cardiac failure patients.

## Introduction

The cardiovascular diseases and the related complications are one of the causes of mortality in both the developed and developing countries including Iran [**1**]. Cardiac failure is a chronic and advancing problem constituting the most common reason for hospital stay in patients of over 60 years [**2**]. Based on some reports, one third of 55-year-old individuals would be affected by this health condition in the later course of life. Unfortunately, only 35% of the cardiac failure patients survive up to 5 years after diagnosis [**3**]. The characteristic feature of this disorder is the inability of the heart to respond to the needs of the tissues due to the reduced power of the left ventricle in pumping enough blood leading to its progressive degenerative functioning in the absence of effective treatment intervention [**4**, **5**].

 This cardiac failure often causes some changes in life style and low quality of the patients’ life [**6**]. Regarding the crippling effects of the nuance experienced by these patients, the results of the study showed that 73.3% of the patients feel a kind of constraint and isolation. Also, 63.3% of them had no expectancy of future life and had accepted that they could have no activity or care of themselves. 66.6% of the patients experienced repeated hospitalization and mentioned lack of sufficient knowledge about self-care and inability to do physical activity as the main causes of this problem [**7**]. This brings about significant therapeutic, social, and financial problems for the patient [**8**]. Regarding the importance of promoting life quality in heart defect patients and the significant role that rehabilitation plays in preventing the cardiac mortality and morbidity, giving more attention to the concept of life quality and the role of rehabilitation is rendered as mandatory. Promotion of life quality is one of the major goals of cardiac patients. The increased need and inclination to cardiac surgery necessitates the examining of postoperative life quality. There are various definitions with regard to health-related life quality. One definition related life quality to the physical, psychological, and social aspects of life being obviously are affected by the individual’s everyday activities [**9**]. Rehabilitation can slow down or control the course of heart failure progress, improve the symptoms of the disease, promote exercise tolerance and life quality, and ultimately lead to a decreased mortality [**10**]. The rehabilitation program is a wide and comprehensive program which, in addition to physical activity and exercises, includes psychological consultation, nutritional consultation, blood pressure control, blood fat control, blood sugar control, stopping smoking, etc. Although cardiac rehabilitation is a basic component of heart patient’s care and is considered as a priority in the countries with a high prevalence of coronary heart disease and cardiac failure [**11**], the number of patients participating in the rehabilitation program is less than expected even in the developed countries [**12**]. The purpose of the present study was to determine the effect of rehabilitation on the heart patients’ life quality. In the case of the existence of a significant difference between the life quality of these patients, the findings may be used by patients, physicians, and other interested personnel to give more importance to these activities, and modify the attitudes of physicians, nurses, and other personnel so that more practical attention will be given to the issue. In this way, we can encourage the patients, provide facilities, and increase the number of patients referring to rehabilitation centers.

## Materials and Methods

This was a before and after study in which heart failure patients with LVEF ≤ %40 referring to the rehabilitation unit participated with the physician’s order after obtaining the approval of the Committee of Ethics in the Cardiovascular Research Center at Yazd University of Medical Sciences. The patient’s informed written consent for participation was obtained. The data collection instrument was a questionnaire consisting of two parts: the first part included the patient’s demographic information and other data as echocardiography, exercise test, blood test (sugar, fat profile, and hepatic tests), angiography, drug consumption history, and patient hospitalization history. The second part was the Life Quality SF-36 Form. The SF-36 Questionnaire included 36 items organized in 8 components including physical performance, limitation of physical performance, bodily pain, general well-being, vital force, social functioning, limitation of psychological role and psychological health. 

 The rehabilitation program included 24-32 sessions. The patients referring to the rehabilitation unit registered to enter the program. Demographic information, background data, and life quality form was filled in at the beginning of the program. The patients’ risk level was determined based on symptoms, exercise test, echocardiography, fat profile tests, and blood sugar tests. The patients underwent psychological and nutritional consultation. The number of consultation sessions required for each patient was planned on the basis of the patient’s needs.The exercise program was planned based on the patient’s risk level. Each exercise session consisted of 20 min of warming up, 20-40 min of aerobic exercises (bicycle, treadmill, and arm ergo meter) based on the patient’s status and with a severity of 60-80% of the patient’s maximal heart rate, 5 min of cooling down, and 20 min of relaxation. The patients were examined daily before of the program started for clinical symptoms and signs, vital signs, and in diabetic patients for blood sugar and the patients’ physical activities were planned. The blood fat, sugar profile, and anthropometry tests were controlled again in the middle of the program and if necessary, the patients repeated the nutritional consultation. At the end of the program, repeated visit, exercise test, echocardiography, blood fat and sugar profile tests, and Life Quality SF-36 Form were performed again. The data were analyzed and compared using SPSS. The criteria for patients’ participation in the program included being a heart failure patient with LVEF ≤%40, an indigenous resident, WNL hemodynamic symptoms, and lack of sensory, motor, and auditory disturbances. The exclusion criteria included chest pain, electrocardiographic changes, patient’s self report on the lack of tolerance, and acute life-threatening hemodynamic changes in the patient. The patients’ before and after rehabilitation data were collected and analyzed using SPSS.

## Results

A total of 34 CHF (Congestive Heart Failure) patients participated in this study. Before the rehabilitation program, the required data were collected using the SF-36 Life Quality Questionnaire and the same information were obtained again after the program. The data were analyzed and compared with SPSS. The sample consisted of 29 (85.3%) males and 5 (14.7%) females. The mean of the patients’ EF was 32.58±6.30 and the mean age of the patients was 50.09 years with a range of 39-84. The findings of the study showed that the mean of the total score of life quality of the sample was 43.48±10.19 before the program and 71.10±13.47 after the program. The difference between the patients was measured with paired T-test which was statistically significant at P<0.001. The mean scores of the aspects of patients’ life quality are presented in (**Table 1**). As the table shows, the results of the comparison of life quality score of the patients before and after the rehabilitation program indicated that there has been an improvement in the following: physical performance or functioning, limitation of physical health, limitation of psychological health, energy, social functioning, and physical pain after rehabilitation with a statistically significant difference. However, this difference was not significant for E.W.B and general health.

**Table 1:**
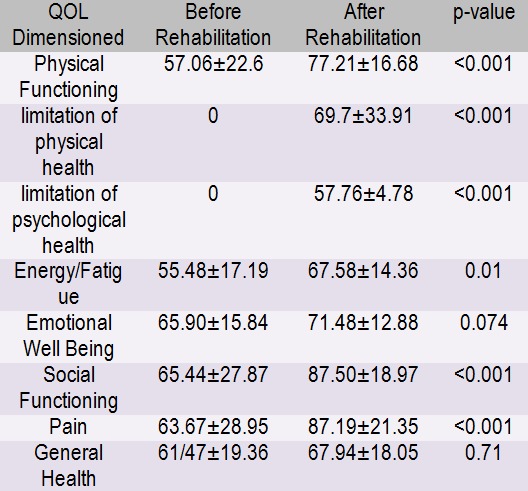
The mean scores of the aspects of life quality before and after Rehabilitation)

## Discussion

The cardiac rehabilitation programs are used as comprehensive plans for improving the life quality of coronary heart disease and CHF patients. The purpose of the study was to investigate the efficacy of the rehabilitation program on the CHF patients’ life quality. Our findings demonstrated that the program significantly improved the total score of life quality and also the scores of six out of eight aspects of life quality. Regarding the aspects of psychological health and general health, there was a relative improvement, though, the difference was not significant. The analysis of the tables obtained from the SF-36 questionnaire revealed the improvement in the physical and psychological aspects. It seems that the improvement in the capacity of cardiopulmonary functioning which results from regular physical activity affects the better understanding of health and also the improvement of life quality. This is consistent with the findings of other studies as they, too, emphasize the efficacy of the educational-exercise programs on the improvement of life quality of CHF patients [**12**-**14**]. In contrast, there are some studies that contradict these findings. For example, Wijkstra et al. found no statistically significant correlation between the rehabilitated patients regarding the physiological and life quality data [**15**]. A similar study was carried out in Turkey by D. G. Kulcu et al. aiming at investigating the effect of cardiac rehabilitation of life quality, anxiety, and depression in 60 CHF patients. Contrary to our findings, the results of this study showed no statistically significant effect of cardiac rehabilitation on the improvement of life quality of CHF patients [**16**]. This difference can be accounted for by the following: First, the sample of D. G. Kulcu et al.’s study was mostly made out of aged, retired, and housekeepers who led a sedentary living. On the other hand, the questionnaire selected for measuring the life quality of these patients was not the SF-36 Form. Rather, they used HQOL including items as sexual activity, work status, and social life which may not be suitable for evaluating the life quality of this age group. Our study used the SF-36 questionnaire and the mean age of the patients was younger. Furthermore, the study by D. G. Kulcu et al. used only the physical activity in cardiac rehabilitation and ignored aspects as psychological counseling, nutritional consultation, etc. which have the potential to reduce the efficacy of cardiac rehabilitation on the improvement of life quality. The other debatable factor contributing to the efficacy of cardiac rehabilitation on life quality in this and similar studies is the examination of the efficacy of long term or short term programs. May be the inconsistency of our results with those of the other study is attributable to the point that the other study measured the short term findings (4 weeks).The study by Kavanagh et al. aimed at investigating the life quality and cardiopulmonary functioning of CHF patients. The findings of this study revealed that there was an initial improvement in the aerobic capacity and hemodynamic symptoms in 4 weeks. Yet, the maximal time required for reaching the final response in the cardiopulmonary and physical variables was 16-26 weeks. Also, the expected improvement in the aspects of life quality appeared in the long run [**17**]. In some cases, a strong correlation was found between life quality and other hemodynamic imbalances and lack of exercise tolerance [**16**]. The review study evaluating the life quality of CHF patients revealed that the improvement in the exercise capacity of CHF patients was not always and consistently associated with the improvement in all aspects of life quality [**18**]. Even if the study conducted by D. G. Kulcu reveal an insignificant improvement in the physical aspect of life quality, they show no improvement in the total score of life quality. This demonstrates that physical activity may only improve the physical activity of life quality.

## Conclusion 

Briefly, although some studies indicate the lack of efficacy of cardiac rehabilitation on improving the life quality of CHF patients, most studies including ours demonstrate the positive effect of cardiac rehabilitation on improving the life quality of this population of patients. It is advisable to include cardiac rehabilitation as part of the therapeutic programs of congestive heart failure.

Acknowledgements Authors would like to thank individuals who assisted in the performance of the studies and/or in the preparation of the manuscript. 
